# Vesicular MicroRNA as Potential Biomarkers of Viral Rebound

**DOI:** 10.3390/cells11050859

**Published:** 2022-03-02

**Authors:** Wilfried Wenceslas Bazié, Julien Boucher, Isidore Tiandiogo Traoré, Dramane Kania, Diane Yirgnur Somé, Michel Alary, Caroline Gilbert

**Affiliations:** 1 Axe de Recherche Maladies Infectieuses et Immunitaires, Centre de Recherche du CHU de Québec-Université Laval, Quebec City, QC G1V 4G2, Canada; julien.boucher.2@ulaval.ca; 2 Programme de Recherche sur les Maladies Infectieuses, Centre Muraz, Institut National de Santé Publique, Bobo-Dioulasso 01 BP 390, Burkina Faso; tiandiogo2002@yahoo.fr (I.T.T.); draka3703@yahoo.fr (D.K.); some.diane@yahoo.fr (D.Y.S.); 3 Département de Santé Publique, Institut Supérieur des Sciences de la Santé, Université Nazi Boni, Bobo-Dioulasso 01 BP 1091, Burkina Faso; 4 Axe de Recherche Santé des Populations et Pratiques Optimales en Santé, Centre de Recherche du CHU de Québec-Université Laval, Quebec City, QC G1S 4L8, Canada; michel.alary@crchudequebec.ulaval.ca; 5 Département de Médecine Sociale et Préventive, Faculté de Médecine, Université Laval, Quebec City, QC G1V 0A6, Canada; 6 Institut National de Santé Publique du Québec, Quebec City, QC G1V 5B3, Canada; 7 Département de Microbiologie-Infectiologie et d’Immunologie, Faculté de Médecine, Université Laval, Quebec City, QC G1V 0A6, Canada

**Keywords:** extracellular vesicles, HIV-1, viral replication, microRNA, miR-29a, miR-146a, miR-155, biomarker

## Abstract

Changes in the cellular microRNA (miRNA) expression profile in response to HIV infection, replication or latency have been reported. Nevertheless, little is known concerning the abundance of miRNA in extracellular vesicles (EVs). In the search for a reliable predictor of viral rebound, we quantified the amount of miR-29a, miR-146a, and miR-155 in two types of plasma extracellular vesicles. Venous blood was collected from 235 ART-treated and ART-naive persons living with HIV (85 with ongoing viral replication, ≥20 copies/mL) and 60 HIV-negative participants at five HIV testing or treatment centers in Burkina Faso. Large and small plasma EVs were purified and counted, and mature miRNA miR-29a, miR-146a, and miR-155 were measured by RT-qPCR. Diagnostic performance of miRNA levels in large and small EVs was evaluated by a receiver operating characteristic curve analysis. The median duration of HIV infection was 36 months (IQR 14–117). The median duration of ART was 34 months (IQR 13–85). The virus was undetectable in 63.8% of these persons. In the others, viral load ranged from 108 to 33,978 copies/mL (median = 30,032). Large EVs were more abundant in viremic participants than aviremic. All three miRNAs were significantly more abundant in small EVs in persons with detectable HIV RNA, and their expression levels in copies per vesicle were a more reliable indicator of viral replication in ART-treated patients with low viremia (20–1000 copies/mL). HIV replication increased the production of large EVs more than small EVs. Combined with viral load measurement, quantifying EV-associated miRNA abundance relative to the number of vesicles provides a more reliable marker of the viral status. The expression level as copies per small vesicle could predict the viral rebound in ART-treated patients with undetectable viral loads.

## 1. Introduction

Human immunodeficiency virus (HIV) causes a chronic infection characterized by reservoirs of latent retroviruses, making its eradication in the host very difficult [1]. Antiretroviral therapy (ART) suppresses virions replication, keeping it below detectable levels or at a low level of viremia in conjunction with small numbers of infected cells [1,2,3]. Maintaining this suppression requires long-term adherence to ART with regular monitoring of viremia. In some patients, viral rebound occurs. It is defined as a persistent detectable level of HIV RNA arising in the blood after a period of suppression. Rebounds and persistent episodes of low-level detectable viremia are associated with a greater risk of subsequent virological failure, drug resistance, and impaired immune status [4,5,6].

In the absence of reliable early viral rebound indicators, various mathematical models and biomarkers have been studied as predictors of the time to viral rebound after interrupting ART [7,8]. While no strong evidence for any biomarker other than total HIV-1 DNA has been found, the persistence of low-level viremia has emerged as a factor in subsequent virological failure and is, therefore, a development that needs to be managed and prevented [9]. There is an urgent need to find a quantitative biomarker of viral rebound that is more accessible than total HIV-1 DNA and provides a better understanding of the mechanisms underlying viral rebound.

The discovery of secreted extracellular vesicles (EVs) and their role as intercellular messengers sheds new light on several physiological and pathological processes [10,11]. EVs are a heterogeneous group of circulating membranous bodies that vary in size and function, depending on their origin and biogenic pathway. They contain complex sets of functional molecules, including proteins, lipids, DNA, mRNA, and microRNA (miRNA) contents [10,11], which reflect the cell type of origin and suggest their investigation as a source of novel biomarkers in infectious diseases and cancer detection. Accumulating evidence suggests that miRNA expressed in EVs has diagnostic value as a liquid biopsy for diagnosis, prognosis, and monitoring several cancers [12,13,14]. Our previous studies have shown that, in patients living with HIV, large and small plasma EVs are loaded differentially with miRNA and may be helpful as biomarkers of immune activation [15,16].

At least one study suggests that HIV-1 modulates EV secretion through its negative regulatory factor (Nef) protein [17]. It was later demonstrated that, in cells infected with HIV-1 or human T lymphotropic virus type 1, the production of EVs containing viral materials precedes viral replication [18]. EVs can regulate the persistence and reactivation of HIV reservoirs through the deregulation of specific pathways that play a significant role in HIV replication [19,20].

Changes in cellular miRNA expression profiles in response to HIV infection have been reported [21]. The miRNA species involved in HIV infection can be divided into those that target HIV transcripts directly and those that affect the viral cycle through the regulation of host cellular factors [22]. Many miRNAs such as the miR-29 family and miR-155 have been associated with HIV replication and latency [23,24,25]. MiR-29a has been found very abundant in HIV-1-infected human T lymphocytes [23], and inhibiting it enhances HIV-1 viral production and infectivity, whereas the expression of miR-29 mimics suppresses viral replication. Differential expression between reactivated and latent cells has been observed for miR-155 and miR-146a, raising the possibility that specific miRNAs expressed abundantly in reactivated cells counter viral activation and perhaps promote a return to latency during transient virus production [25].

The idea that HIV replication modulates EV secretion and induces modification of cell miRNA expression led us to wonder if the EV miRNA content might predict viral rebound in patients on ART. In this article, we investigate the miR-29a, miR-146a, and miR-155 expression levels in large and small plasma EVs purified from HIV patients with and without viral replication and evaluate the potential of this measurement as a rapid clinical test for identifying viremic patients.

## 2. Materials and Methods

### 2.1. Study Subjects

HIV-infected participants were recruited by referral from five follow-up centers in Bobo-Dioulasso and Ouagadougou (Burkina Faso). These included the Yerelon clinics (Centre Muraz) of both towns, specialized in female sex worker follow-up, the Association African Solidarité (AAS) center for the follow-up of men who have sex with men, and dedicated follow-up day hospital centers CHU Sourô Sanou (Bobo-Dioulasso) and CHU Yalgado Ouédraogo (Ouagadougou). The HIV-negative participants were recruited in the Yerelon clinics, which also offer HIV testing. 

### 2.2. Quantitation of HIV-1 RNA, CD4 and CD8 T Lymphocytes

The HIV-1 viral load was measured using the COBAS^®^ AmpliPrep /COBAS^®^ TaqMan^®^ Real-Time PCR assay (TaqMan, Roche Diagnostics, Mannheim, Germany), which targets two highly conserved regions of the HIV-1 genome and has a detection limit of 20 copies/mL.

Absolute counts of CD4+ T and CD8+ T lymphocytes were obtained using a BD FACSCount™ System flow cytometer (Becton Dickinson, San Jose, CA, USA).

### 2.3. Purification of Extracellular Vesicles

Purification of EVs was performed as described previously [15,16]. Briefly, blood obtained by venipuncture with citrate as an anticoagulant was centrifuged for 10 min at 400× *g* at room temperature. The plasma was centrifuged for 10 min at 3000× *g* to obtain platelet-free-plasma and stored at −80 °C until analysis. Thawed platelet-free plasma (250 µL) was treated with proteinase K (1.25 mg/mL, Ambion™, Thermo Fisher Scientific, Waltham, MA, USA) for 10 min at 37 °C. This pretreatment of plasma with proteinase K is described to critically reduce the amount of non-EV proteins (albumin and the apolipoproteins A-1 and B), which can be co-purified with EV. This digestion step destroys proteins and their cargo (mRNA and miRNA), and they were removed with the washing step. Large EVs were purified by centrifuging proteinase-K-pretreated plasma for 30 min at 17,000× *g* at room temperature. The supernatant was mixed with 63 µL of ExoQuick-TC™ (SBI via Cedarlane, Burlington, ON, Canada) in an Eppendorf tube and maintained at 4 °C overnight. The large EV pellet was resuspended in 250 µL of microfiltered (0.22-µm pore size membrane) 1x phosphate-buffered saline (WISENT Bioproducts, Saint-Jean-Baptiste, QC, Canada) and centrifuged for 30 min at 17,000× *g*. The supernatant was discarded, and the washed large EV pellet was resuspended in 250 µL of PBS and kept at 4 °C. Small EVs were obtained from the ExoQuick-TC™ precipitation after centrifuging for 30 min at 1500× *g*. The ExoQuick precipitate was centrifuged for 30 min at 1500× *g*. The supernatant was discarded, and the pellet was washed in PBS and centrifugated for 5 min at 1500× *g* to obtain small EVs, which were resuspended in 250 µL of PBS by vortex mixing and kept at 4 °C. The size of the EV was determined by hydrodynamic radius measurements using a Zetasizer Nano S (Malvern Instruments, Ltd., Malvern, United Kingdom). This technique is based on the light scattering intensity due to the Brownian motion of EVs, characterized by a diffusion constant [26]. The size distribution is obtained from the distribution of diffusion constants using the Einstein–Stokes equation [26]. The measurements were made at a fixed position with an automatic attenuator and at a controlled temperature. One hundred microliters of EV suspension were used for each sample, and two measurements were averaged. We have submitted all relevant data of our experiments to the EV-TRACK knowledgebase (EV-TRACK ID: EV220124) [27].

### 2.4. EV Flow Cytometry Analysis

Purified EVs were stained with the lipophilic fluorescent carbocyanine dye DiD (DiIC18(5) solid: 1,1′-dioctadecyl-3,3,3′,3′-tetramethylindodicarbocyanine 4-chlorobenzenesulfonate salt (Invitrogen™, Carlsbad, CA, USA), and the vesicular or cell-permeable dye CFSE (carboxyfluorescein diacetate succinimidyl ester, Invitrogen™, Carlsbad, CA, USA). DiD and CFSE were prediluted, respectively, 1/100 and 1/500 with filtered (0.22 µm) PBS 1X + EDTA (100 µM for the final concentration). Forty microliters of DiD diluted solution (final dilution 1/200, 1 µg/mL) was added to 10 µL of EV suspension. After 5 min at 37 °C, 50 µL of CFSE (diluted 1/1000, 1 µg/mL) were added. After 15 min (37 °C), the staining was fixed by adding 0.02% Pluronic F-127 (Invitrogen™, Carlsbad, CA, USA) solution. Next, 100 µL of 4% paraformaldehyde (Fisher scientific™, Ottawa, CA, USA) solution was added and incubated for 20 min, and the tube was completed to 400 µL with 200 µL of filtered PBS 1X. Then, 5 µL of 15-µm count beads (Polybead^®^ Microspheres 15 µm, Polysciences, Inc., Warrington, PA, USA) were mixed in by vortex. A flow cytometry method described previously [15,16] was used to count EVs in a FACS Canto II Special Order Research Product cytofluorometer equipped with forward scatter coupled to a photomultiplier tube (FSC-PMT) with the “small particles option” (BD Biosciences, Franklin Lakes, NJ, USA). Gating strategies for EV identification and analysis are described elsewhere [16].

### 2.5. MicroRNA Quantification

EV suspension (100 µL) was diluted 3:1 in TRIzol LS (Ambion™, Life Technologies, Carlsbad, CA, USA) and maintained at −80 °C. Total RNA was extracted, mixed with 10 µL of diethylpyrocarbonate water, and quantified in 1 µL using a BioDrop-μLITE kit (Isogen Life Science, Utrecht, The Netherlands). After treatment with RNase-free DNase I (Ambion™ Life Technologies), 100 ng of RNA was reverse-transcribed using a HiFlex miScript RT II kit according to the manufacturer’s instructions (Qiagen, Hilden, Germany). Mature miR-29a (#MS00003262), miR-146a-5p (#MS00006566), and miR-155-5p (#MS00031486) were detected by quantitative polymerase chain reaction (RT-qPCR) using an miScript primer assay kit and miScript SYBR Green PCR kit (Qiagen). Mature microRNA as cDNA was amplified in a CFX384 Touch Real-Time PCR Detection System (Bio-Rad, Hercules, CA, USA) using 40 cycles of 94 °C for 15 s, 55 °C for 30 s, and 72 °C for 30 s. Reaction specificity was confirmed using the melt curve procedure (65–95 °C, 0.5 °C per 5 s) at the end of the amplification protocol according to the manufacturer’s instructions. A microRNA control (miRTC control # MS00000001) was used in conjunction with the miScript II RT Kit to ensure the quality of the reverse transcription during the qPCR step. A standard curve was used for the absolute quantification of microRNA. Quantifying miRNA was expressed as copies per µg of RNA and copies per vesicle, as described previously [15].

### 2.6. Transmission Electron Microscopy

Briefly, large and small EV pellets purified as described above were suspended with glutaraldehyde 2.5% (Sigma-Aldrich, St. Louis, MO, USA) in 0.1-M sodium cacodylate (Sigma-Aldrich, St. Louis, MO, USA) buffer, pH 7.4. Ten microliters of the EV sample were pipetted onto a nickel grid with carbon-coated formvar film and incubated for 10 min. Excess liquid was removed by blotting. The grids were stained for 2 min with 2% uranyl acetate solution. Images were acquired using a FEI Tecnai Spirit G2 (FEI, Eindhoven, The Netherlands) transmission electron microscope equipped with a bottom-mounted CCD camera at 80 kV.

### 2.7. Statistical Analysis

All analyses were performed with GraphPad Prism 8 (GraphPad Inc, San Diego, CA, USA) and RStudio Version 1.4.1103 (Integrated Development for R. RStudio, PBC, Boston, MA, USA, 2020, http://www.rstudio.com/) to build correlation matrices and principal component analyses. Participant demographic and clinical characteristics were presented as a proportion or median with an interquartile range (IQR) and tabulated (Table 1 and Table S1). Initial tests of normality and log normality indicated that the EV count and miRNA content expressed as copies/µg and copies/vesicle both fit a lognormal distribution. All values were therefore transformed to logarithms. Data were then analyzed assuming a Gaussian distribution using parametric tests, and ordinary one-way ANOVA corrected for multiple comparisons using the Tukey test for three or more group comparisons. In all graphs, the results are presented as the geometric mean with geometric standard deviation factor. Pearson parametric correlation tests were performed to build correlation matrices in R. The diagnostic value of the EV miRNA content was evaluated using receiver operating characteristic (ROC) curves. Analyses were performed using the Wilson/Brown method, and the results were tabulated. A *p*-value less than 0.05 was considered statistically significant.

## 3. Results

### 3.1. HIV-1 Replication Could Be Induces the Production of Large EVs

**Figure 1:**
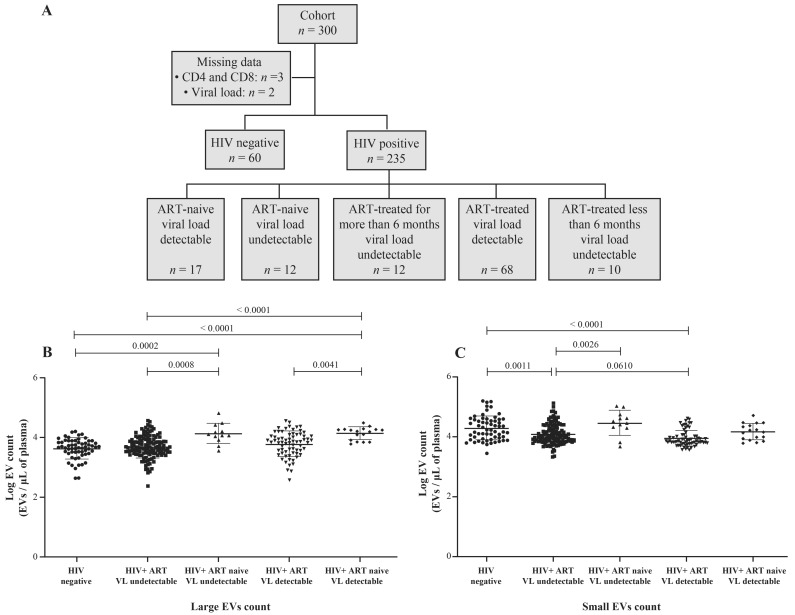
Flow chart of study participant categorization (**A**). Counts of purified large (**B**) and small (**C**) plasma EVs by flow cytometry using DID and CFSE staining. Study participants were HIV-negative, HIV-positive on antiretroviral therapy (ART) for more than six months, or HIV-positive and ART-naïve, with an undetectable or detectable viral load (VL). An ordinary one-way ANOVA corrected for multiple comparisons using the Tukey test was used to compare groups.

HIV-1 replication in vitro induces the production of EVs [28], and heterogeneous EV populations found in plasma from ART-naive people living with HIV (PLWH) are more abundant than in successfully treated ART patients [29]. This study focused on two categories of EVs, called large and small (Figure S1). The demographic and clinical characteristics of the participants are summarized in Table 1 and Table S1. The median age was 36 years (IQR 30–44) in the HIV+ group (*n* = 235) and 27 years (IQR 22–32) among the uninfected participants (control, *n* = 60). The median CD4 T cell counts were, respectively for the control and HIV+ group, 992 (IQR 794–1276) and 466 (IQR 330–696) per µL, and the median CD8 T cell counts were 584 (IQR 447–715) and 770 (IQR 543–1089) per µL, giving median CD4/CD8 ratios of 1.8 (IQR 1.4–2.1) and 0.6 (IQR 0.4–1.0). In PLWH participants, 40.9% were female sex workers, and 17.0% were men who have sex with men. The median duration of HIV infection was 36 months (IQR 14–117), and 87.7% of HIV+ participants were ART-treated for 34 months (IQR 13–85). HIV-1 RNA was undetectable (viral load < 20 copies/mL) in 63.8% of these persons, and the median detectable viremic load was 30,032 copies/mL (IQR 108–33,978). Participants’ HIV status, ART use, and viral load are summarized in Figure 1A.

Our previous observations of small EVs being the more abundant category in plasma [15,16] are corroborated (Figure 1B,C). EVs are overall more abundant in ART-naive viremic or nonviremic participants than in the corresponding ART-treated patients (Figure 1B,C and Figure S2B,C). A significant difference in small EV abundance is noted between viremic ART patients and nonviremic (successfully treated) ART patients (Figure S2C). These results show that large plasma EVs accumulate during HIV-1 replication and that ART decreases EV abundance. More importantly, small EV abundance decreases significantly during ART in viremic patients with over 1000 copies/mL.

### 3.2. MicroRNA Content of Large versus Small EVs

**Figure 2:**
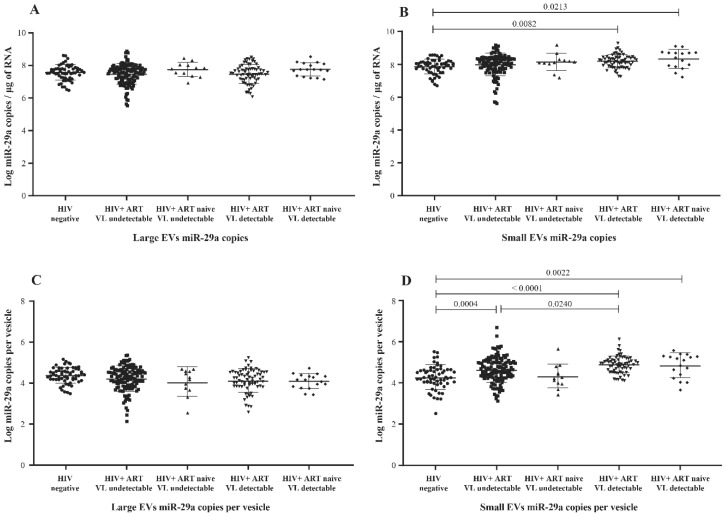
Mature miR-29a content expressed as copies per µg of total RNA in large (**A**) and small (**B**) plasma EVs and expressed as copies per large vesicle (**C**) and copies per small vesicle (**D**). See the flow chart in Figure 1 for study participant group descriptions. Values are log10-transformed. An ordinary one-way ANOVA was used with the Tukey test for between-group multiple comparisons.

In previous studies using peripheral blood mononuclear cells, the concentration of miR-29a was found inversely correlated with viremia, and its overexpression has been associated with the inhibition of viral replication and thus appears to force HIV-1 into latency [23,24,30]. Comparing nonviremic HIV+ patients relative to the control (HIV-) participants, we found no significant difference in miR-29a expressed as copies per µg RNA or copies per large vesicle (Figure 2A,C). In contrast, miR-29a copies per µg RNA were increased significantly in small EVs of viremic patients compared to the control group (Figure 2B), as were copies per vesicle (Figure 2D). In addition, copies per vesicle were significantly increased in viremic compared to nonviremic ART-treated patients (Figure 2D). This latter comparison held properly over different levels of viremia (Figure S3D). These data show altogether that HIV-1 replication during ART induces the overexpression of miR-29a in small plasma EVs.

**Figure 3:**
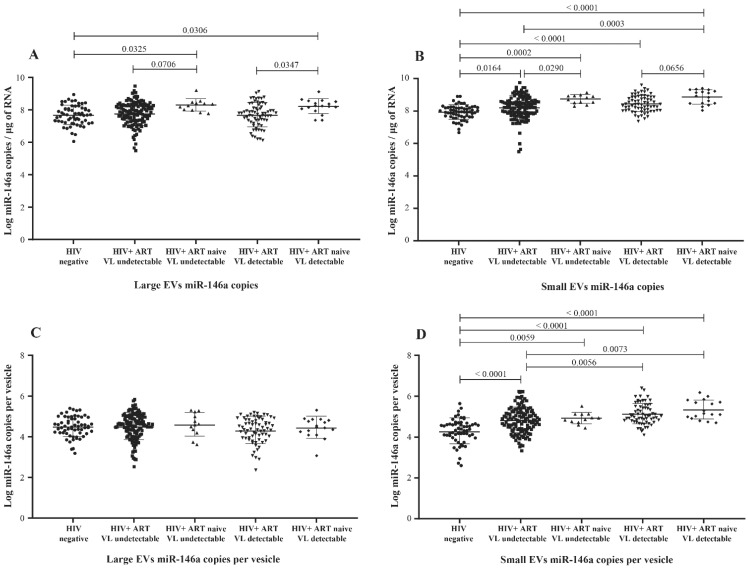
Mature miR-146a content expressed as copies per µg of total RNA in large (**A**) and small (**B**) plasma EVs and expressed as copies per large vesicle (**C**) and copies per small vesicle (**D**). See the flow chart in Figure 1 for study participant group descriptions. Values are log10-transformed. An ordinary one-way ANOVA was used with the Tukey test for between-group multiple comparisons.

MicroRNA 146a production is an established marker of inflammation [30,31], a significant concern in managing HIV infection. Copies of miR-146a in large and small plasma EVs were more abundant in HIV+ participants than in the control group (Figure 3A,B) and more abundant in viremic than nonviremic persons when small EVs are compared (Figure 3B,D), especially when the viral load is at least 1000 copies per mL (Figure S4B,D). ART was associated with a significant decrease in miR-146a in large and small EVs (Figure 3A,B). These results show that miR-146a increases in plasma EVs of HIV+ patients and that viral replication could be potentiating this response in small EVs.

**Figure 4:**
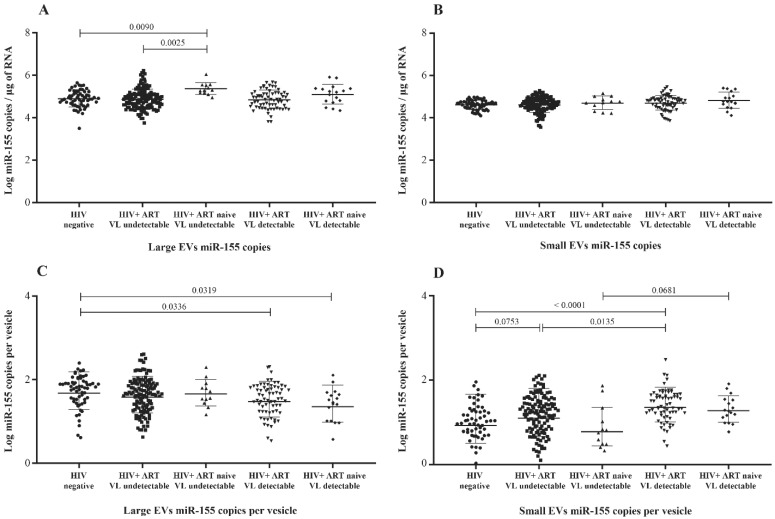
Mature miR-155 content expressed as copies per µg of total RNA in large (**A**) and small (**B**) plasma EVs and expressed as copies per large vesicle (**C**) and copies per small vesicle (**D**). See the flow chart in Figure 1 for the study participant group descriptions. Values are log10-transformed. An ordinary one-way ANOVA was used with the Tukey test for between-group multiple comparisons.

Previous studies have shown elevated levels of miR-155 in plasma, peripheral blood mononuclear cells, CD4 T and CD8 T cells, and EVs from HIV-1-infected patients [29,32,33,34]. Here, we compared its copy numbers in large and small plasma EVs purified from viremic and nonviremic HIV+ patients, as well as uninfected persons. Unlike miR-29a and miR-146a, miR-155 copies per µg of RNA were more abundant in large EVs from ART-naive nonviremic-infected patients, even compared to the ART-treated group (Figure 4A). Its expression in large and small vesicles does not appear to be proportionate to viremia (Figure S5A,B). Copies per large vesicle were decreased in viremic patients compared to control participants (Figure 4C and Figure S5C), whereas copies per small vesicles varied among the participant groups (Figure 4D) but were not proportionate to viremia (Figure S5D). These results nevertheless showed patterns of the differential expression of miR-155 in large and small EVs associated with viremia.

### 3.3. Correlation between EV MicroRNA Content and Demographic/Clinical Parameters

**Figure 5:**
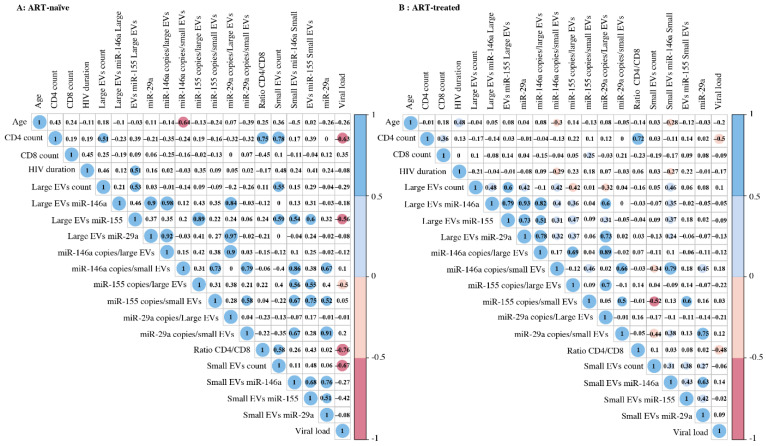
Correlation matrix between the different variables of ART-naive (**A**) or ART-treated (**B**) study participants with viremia. The sizes and colors of the circles indicate the strength of the correlation, and the colored boxes indicate significant correlation at the threshold of 0.05. A two-tailed Pearson correlational test was computed between variables.

Correlograms (Figure 5 and Figure S6) summarize the relationships between our measurements. MiR-155 copies per µg RNA or large EV were correlated significantly and inversely with viral load over 1000 copies per mL of ART-naive (Figure 5A and Figure S6B). In contrast, miR-29a and miR-146a copies in small EVs were positively correlated with the viral load in ART patients with over 1000 viral copies/mL (Figure S6C). An inverse correlation between age and miR-146a expression in small EVs was observed. As expected, the HIV-1 viral load was correlated inversely with the CD4 T-cell count and CD4/CD8 ratio (Figure 5). The number of small EVs was positively correlated with the CD4 T-cell count and negatively with the viral load in ART-naive patients (Figure 5A). Although the correlation between miRNA content and viral load appears to be strong, a significant discriminator between the viremic status groups remains identified.

### 3.4. Principal Component Analysis of the EV Measurement/Viral Replication Association

**Figure 6:**
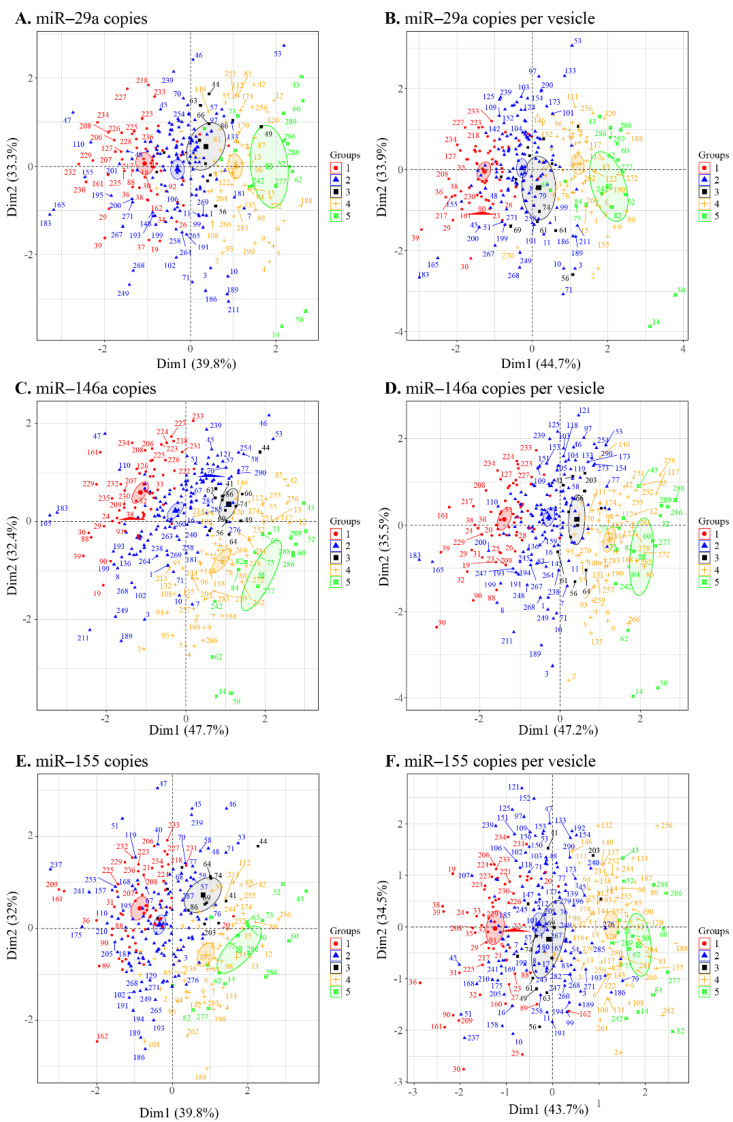
Principal component analysis of the microRNA content of large and small EVs in individuals in the five groups ((1) HIV-negative, (2) HIV+ ART-treated nonviremic, (3) HIV+ ART–naïve nonviremic, (4) HIV+ ART-treated viremic, and (5) HIV+ ART–naïve viremic). The miRNAs miR-29a (**A**), miR-146a (**C**), and miR-155 (**E**) contents expressed as copies per µg of RNA and expressed as copies per vesicle for miR-29a (**B**), miR-146a (**D**), and miR-155 (**F**). Ellipses are drawn around the centroids of the clusters, representing 95% confidence intervals.

Given our correlogram results (Figure 5 and Figure S6) showing several correlations between the miRNA contents of large and small EVs and the clinical status, we used a principal component analysis to discriminate between the five patient groups (Figure 1A) based on miRNA copies per µg of the total RNA and copies per vesicle (Figure 6). The levels of miR-29a (Figure 6A,B), miR-146a (Figure 6C,D), and miR-155 (Figure 6E,F) each clearly distinguished between HIV- and viremic HIV+ individuals. The strength of this measurement is apparent in the confidence ellipses and the mean coordinates of the individuals in each group. Copies per vesicle seem to give a better graphical representation of the individuals, showing more excellent proximity of the viremic (ART-treated and ART-naïve) and nonviremic (ART-treated and ART-naive) group mean points. These results suggest that the quantitative measurement of EV-associated miR-29a, miR-146a, and miR-155 might be helpful for the diagnosis of individual viral statuses.

### 3.5. MicroRNA 29a, 146a, and 155 Copies per Small EV and Mir-155 Copies in Large EV as Potential Biomarkers of Viral Rebound 

**Figure 7:**
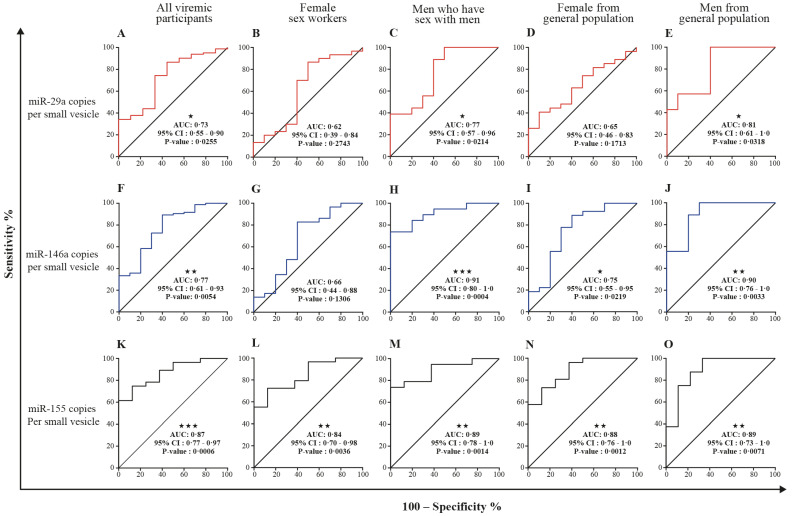
Small EV miRNA content diagnosis performances in the receiver operating characteristics curves analysis. MiRNA per small EVs was used to generate a receiver operator characteristic (ROC) curve analysis to discriminate viremic participants in different populations. The nonviremic patients ART-treated for more than six months and having a CD8 T cell count < 500 cells/µL, CD4 T cell count ≥ 500 cells/µL, and CD4/CD8 ≥ 1 (reference patient) as the controls. The diagnosis performance of miRNA expressed as copies per small vesicle for the discrimination of participants with detectable viral loads in all viremic participants were presented for miR-29a (**A**), miR-146a (**F**), and miR-155 (**K**). The diagnosis performance of miRNA expressed as copies per small vesicle for the discrimination of participants with detectable viral loads in female sex worker group participants were presented for miR-29a (**B**), miR-146a (**G**), and miR-155 (**L**). The diagnosis performance of miRNA expressed as copies per small vesicle for the discrimination of participants with detectable viral loads in men who have sex with men were presented for miR-29a (**C**), miR-146a (**H**), and miR-155 (**M**). The diagnosis performance of miRNA expressed as copies per small vesicle for the discrimination of participants with detectable viral loads in female from general population group were presented for miR-29a (**D**), miR-146a (**I**), and miR-155 (**N**). The diagnosis performance of miRNA expressed as copies per small vesicle for the discrimination of participants with detectable viral loads in men from general population group were presented for miR-29a (**E**), miR-146a (**J**), and miR-155 (**O**). Wilson/Brown method was used to compute the area under a ROC curve.

**Figure 8:**
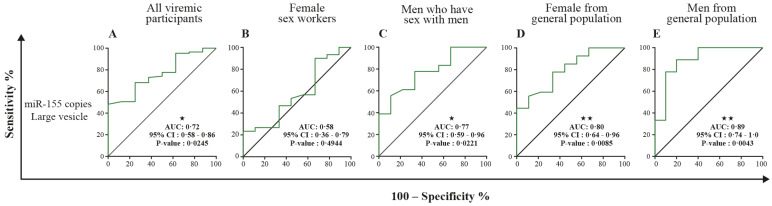
Large EV miR-155 content diagnosis performance in the receiver operating characteristics curves analysis. MiR-155 per large EVs was used to generate a receiver operator characteristic (ROC) curve analysis to discriminate viremic participants in different populations. The nonviremic patients ART-treated for more than six months and having a CD8 T cell count < 500 cells/µL, CD4 T cell count ≥ 500 cells/µL, and CD4/CD8 ≥ 1 (reference patient) undetectable viral load under ART were used as controls. Wilson/Brown method was used to compute the area under a ROC curve. The diagnosis performance of miR-155 copies per µg of total RNA in large for the discrimination of participants with detectable viral were presented for all participants (**A**), female sex workers (**B**), men who have sex with men (**C**), female from general population (**D**), and men from general population (**E**). Wilson/Brown method was used to compute the area under a ROC curve.

Table 2 and Tables S2–S7 show receiver operator characteristic areas under the curve for the predictability of HIV-1 viral rebound based on the EV miRNA content using HIV-negative participants as the control group (Table 2 left column, and Tables S2 and S5), then nonviremic ART-naive patients (Table 2 middle column, and Tables S3 and S6), and finally, nonviremic patients ART-treated for more than six months and having a CD8 T cell count < 500 cells/µL and CD4 T cell count ≥ 500 cells/µL (reference patient) (Table 2 right column, and Tables S4 and S7). Using the reference patient as a control group, miR-29a, miR 146a, and miR155 copies per small EVs (Figure 7) and miR-155 copies in large EVs (Figure 8) discriminated viremic patients. In addition, for low-viremic ART-treated patients (20–1000 copies/mL) and in subgroups of viremic participants (female sex workers, men who have sex with men, and men and women from the general population), miR-29a, miR 146a, and miR-155 copies per small EVs remain the best discriminators of viremic patients (Tables S2–S7). These results confirm that the three miRNA species expressed as copies per vesicle distinguish viremic patients and, more importantly, are the best discriminator of ART-treated low-viremic patients.

## 4. Discussion

HIV infection alters the physiological state of infected and uninfected cells, both of which release EVs containing substances that reflect the current functional state of the cells. Previous studies have shed light on the role of EVs in HIV-1 pathogenesis, in some cases showing that they prime the body to support the viral spread and that blood EVs reflect tissular activation of specific cell types [19,20]. In this study, two types of plasma EVs and their miRNA contents were evaluated as indicators of viral rebound in ART-treated patients. We found that viral replication is associated with the increased production of large EVs and relatively few small EVs based on flow cytometry. The normal function of EVs as a means of sophisticated intercellular communications for numerous purposes, including signaling the presence of pathogens, can be hijacked by viruses to mediate their propagation, which could explain the increased production of EVs in viremic patients. ART drugs reportedly lower EV secretion by HIV-1-infected cells and alter their content [32]. In addition to targeting different stages of the viral life cycle, ART initially brings some reduction in inflammation and immune activation [33] and could explain the decrease in EV production. Our results show that small EVs tend to be less abundant in ART-treated patients than in ART-naive patients, viremic or not.

The release of EVs bearing miRNA reportedly regulates the intracellular miRNA levels directly, and EVs may play a role in cell disposal, since they also contain intracellular components that suggest their other use as garbage bags [34,35]. Significant differences in the content of large versus small plasma EVs from HIV+ subjects confirm observations that HIV infection alters the profile of microRNA expression. Our study is one of the first to compare the expression of miR-29a, miR-146a, and miR-155 in viremic and nonviremic patients in association with large and small plasma EVs. We found that miR-29a is overexpressed in small EVs of viremic patients and that its expression per small EV is the best discriminator of ART-treated patients with low viremia. We also found that miR-146a is overexpressed in large and small plasma EVs of persons with detectable viremia. The expression of miR-155 in copies per vesicle appears to be elevated in small EVs and reduced in large EVs in viremic compared to nonviremic patients. Moreover, this expression was the best discriminator of viremic patients. 

**Figure 9:**
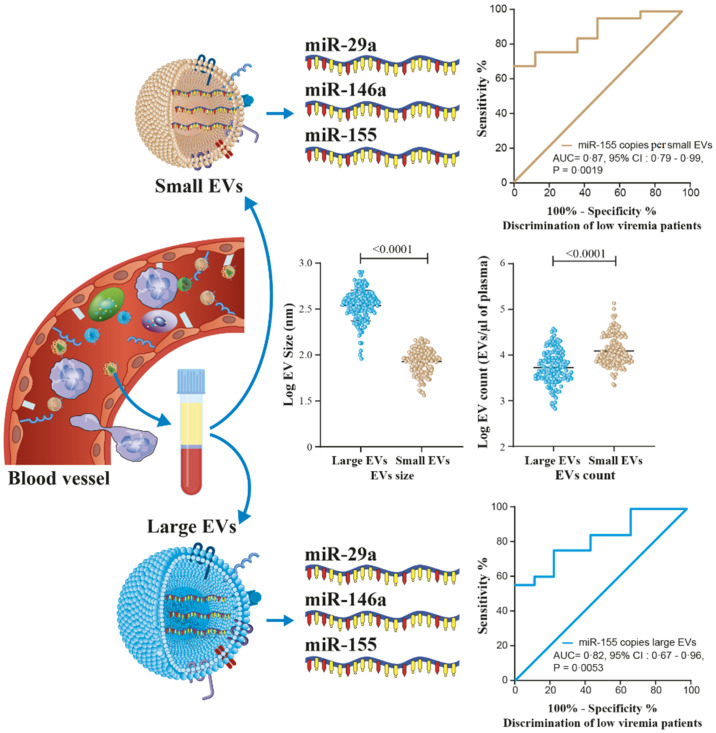
Graphical abstract show that microRNA into large or small EVs in plasma from PLWH may be helpful in the prediction of viral rebound as liquid biopsies for different populations.

Although these three miRNAs are known to influence HIV-1 replication in vitro in several ways, the quantitative relationship remains unclear, and additional mechanisms might exist. MiR-29a is downregulated during HIV replication and correlates inversely with active HIV-1 replication [23,36]. In vitro, it binds to a sequence in the 3′UTR of viral transcripts, silencing viral production and infectivity. Its overexpression downregulates HIV-1 replication and reduces virion infectivity [23,36]. Meanwhile, miR-146a is upregulated in CD4 T-cell-derived EVs from viremic ART-naive HIV+ patients [37]. It is overexpressed in most viral diseases and may be a critical factor in the progression of illness by acting as a brake on the inflammatory response [31]. The relationship between miR-146a and the NF-κB signaling pathway has been reported and is consistent with the involvement of miR-146a in regulating the innate immune response and increased expression in chronic inflammation [31,32,38]. A previous study suggested an increased expression of miR-155 in peripheral blood mononuclear cells and plasma in viremic patients compared to elite controllers or healthy subjects [39]. There is evidence for miR-155 targeting several host-dependent factors involved in post-entry pre-integration events, thereby curtailing HIV-1 infection [38]. MiR-155 has been shown to keep HIV-1 in latency by targeting the TRIM32 transcript and blocking its promotion of HIV-1 reactivation from the latent state [25]. It is also described as a modulator of various aspects and steps of innate and adaptive immune responses [40]. Abundantly expressed in reactivated cells, its expression is required for optimal CD8+ T cell responses to counter viral replication [41,42]. The elevated expression of miR-146 and miR-155 in EVs of viremic patients could serve to inform and protect surrounding cells from HIV infection and help manage host-dependent factors and cellular pathways important for viral replication. The amounts of the three miRNA molecules measured in large and small EVs in this study could be an overall reflection of HIV replication. The reproducibility of our overall results in four subgroups (female sex workers, men who have sex with men, and men and women of the general population) reinforces the biomarker potentials for microRNA in EVs as we monitor ART-treated HIV patients for the earlier detection and management of viral rebound, as illustrated in Figure 9.

## 5. Conclusions

In summary, we reported the differential expression of miR-29a, miR-146a, and miR-155 in large and small plasma EVs from viremic and nonviremic, ART-treated and ART-naive patients and from uninfected persons. The release of EVs from HIV-1-infected cells prior to virion release led us to hypothesize that a clinical analysis of EV contents might provide a biomarker of viral replication in persons living with the virus. We found that miR-29a, miR-146a, and miR-155 expressed as copies per vesicle each discriminate against viremic patients to some extent and could serve this purpose. Due to the cross-sectional nature of our study, a longitudinal follow-up of a cohort of patients is needed to evaluate and validate the suitability of miR-29a, miR-146a, and miR-155 as predictors of viral rebound in ART-treated persons, as well as in an in vitro mechanistic analysis.

## Figures and Tables

**Table 1:** Demographic and clinical characteristics of the study participants.

	Control (*n* = 60)	HIV+ (*n* = 235)	*p*-Value	HIV+			HIV+ Viral Load Detectable		
				Viremic (*n* = 85)	ART > 6 Months, Viral Load Undetectable (*n* = 128)	*p*-Value	ART-Naïve (*n* = 17)	ART-Treated (*n* = 68)	*p*-Value
Male, *n* (%)	14 (23.3)	68 (28.9)	0.3880	28 (32.9)	31 (24.2)	0.1636	6 (35.3)	22 (32.4)	0.8175
Female sex workers: *n* (%)		96 (40.9)		30 (35.3)	63 (49.2)	0.1208	2 (11.8)	28 (41.2)	0.0536
Men who have sex with men: *n* (%)		40 (17.0)		19 (22.3)	20 (15.6)		4 (23.5)	15 (22.1)	
Age (y)	27	36	<0.0001	35	38	0.0553	37	35	0.9577
	(22–32)	(30–44)		(30–42)	(32–45)		(27–4)	(30–42)	
<20	3 (5.0)	5 (2.1)	<0.0001	2 (2.4)	2 (1.6)	0.2670	1 (5.9)	1 (1.5)	0.4993
20–29	34 (56.7)	37 (15.7)		17 (20.0)	16 (12.5)		4 (23.5)	13 (19.1)	
30–39	19 (31.7)	94 (40.0)		37 (43.5)	49 (38.3)		5 (29.4)	32 (47.0)	
40–49	4 (6.6)	70 (29.8)		20 (23.5)	47 (36.7)		5 (29.4)	15 (22.1)	
≥ 50		29 (12.3)		9 (10.6)	14 (10.9)		2 (11.8)	7 (10.3)	
HIV duration (month)	NA	36 (14–117)		31 (8–72)	55 (24–120)	0.0313	8 (1–72)	36 (12–72)	0.9891
CD4 T cells/µL	992 (794–1276)	466 (330–696)	<0.0001	382 (213–561)	513 (386–755)	<0.0001	583 (318–742)	354 (188–507)	0.0860
CD8 T cells/µL	584 (447–715)	770 (543–1089)	<0.0001	841 (573–1049)	736 (536–1093)	0.6081	794 (570–1036)	848 (544–1016)	0.7852
CD4/CD8	1.8 (1.4–2.1)	0.6 (0.4–1.0)	<0.0001	0.5 (0.3–0.8)	0.7 (0.5–1.0)	<0.0001	0.6 (0.4–1.0)	0.4 (0.3–0.7)	0.0185
ART, *n* (%)	NA	206 (87.7%)		68 (80.0%)	NA		NA	NA	
ART duration (month)	NA	34 (13–85)		24 (8–62)	38 (21–96)	0.0231	NA	24 (8–62)	
Undetectable HIV viral load (<20 copies/mL), *n* (%)	NA	150 (63.8)		NA	NA		NA	NA	
HIV-1 viral load (copies/mL)	NA	30,032		30,032	NA		17,330	1446	
	NA	(108–33,978)		(108–33,978)	NA		(3,032–39,416)	(82–24,578)	0.2958

Ranges indicated are interquartile (IQR); NA = not applicable; CD4 and CD8 counts are in cells per µL, HIV and ART refer to human immunodeficiency virus and antiretroviral therapy status.

**Table 2:** Receiver operating characteristic analysis of EV miRNA content as a discriminator of viremic patients versus control.

	Group Used as Control					
	HIV-Negative		ART-Naïve, Undetectable Viral Load		Reference Patients	
	AUC (95% CI)	*p*-Value	AUC (95% CI)	*p*-Value	AUC (95% CI)	*p*-Value
miR-29a copies, large EVs	0.52 (0.43–0.62)	0.6006	0.63 (0.47–0.78)	0.1548	0.58 (0.38–0.77)	0.4274
miR-29a copies, small EVs	0.71 * (0.63–0.80)	<0.0001	0.60 (0.45–0.76)	0.2686	0.50 (0.35–0.65)	0.9600
miR-29a copies per large EV	0.65 (0.56–0.74)	0.0016	0.50 (0.31–0.69)	0.9911	0.54 (0.31–0.76)	0.7099
miR-29a copies per small EV	0.80 (0.73–0.88)	<0.0001	0.88 (0.49–0.97)	<0.0001	0.73 (0.55–0.90)	0.0255
miR-146a copies, large EVs	0.54 (0.44–0.63)	0.4420	0.72 * (0.61–0.83)	0.0133	0.60 (0.43–0.78)	0.2914
miR-146a copies, small EVs	0.81 (0.74–0.88)	<0.0001	0.63 (0.52–0.75)	0.1348	0.66 (0.48–0.83)	0.1082
miR-146a copies per large EV	0.56 (0.46–0.65)	0.2285	0.62 (0.45–0.80)	0.1695	0.54 (0.34–0.74)	0.6801
miR-146a copies per small EV	0.89 (0.83–0.94)	<0.0001	0.68 (0.55–0.81)	0.0502	0.77 (0.61–0.93)	0.0054
miR-155 copies, large EVs	0.51 (0.41–0.60)	0.8914	0.80 * (0.70–0.90)	0.0007	0.76 (0.63– 0.90)	0.0084
miR-155 copies, small EVs	0.63 (0.54–0.72)	0.0093	0.52 (0.35–0.69)	0.8010	0.61 (0.46–0.75)	0.2891
miR-155 copies per large EV	0.68 * (0.59–0.77)	0.0002	0.62 (0.45–0.78)	0.1674	0.52 (0.29–0.75)	0.8367
miR-155 copies per small EV	0.76 (0.68–0.85)	<0.0001	0.97 (0.93–100)	<0.0001	0.87 (0.77–0.97)	0.0006

HIV: human immunodeficiency virus, ART: antiretroviral therapy, AUC: area under the curve (with 95% confidence interval) based on all viremic patients with ≥ 20 copies per mL, and CI: confidence interval. * Values are higher in the control.

## Data Availability

Deidentified participant data from this study and corresponding data dictionary, study protocol, and informed consent documents will be made available to researchers upon request to the corresponding authors. Researchers will be asked to complete a concept sheet for their proposed analyses to be reviewed, and the investigators will consider the overlap of the proposed project with active or planned analyses and the appropriateness of the study data for the proposed analysis.

## Supplementary Materials

The following supporting information can be downloaded at https://www.mdpi.com/article/10.3390/cells11050859/s1: Table S1: Demographic and clinical characteristics of the study participants. Figure S1: Extracellular vesicles characterization and miRNA content. Figure S2: Extracellular vesicle analysis in cytometry. Figure S3: MicroRNA miR-29a content in large and small EVs linked with the viral load status. Figure S4: MicroRNA miR-146a content in large and small EVs linked with the viral load status. Figure S5: MicroRNA miR-155 content in large and small EVs linked with the viral load status. Figure S6: Correlation matrix between measurements on viremic HIV+ patients, ART-naïve HIV patients with a viral load ≥ 1000 copies/mL, ART-treated HIV patients with a viral load ≥ 1000 copies/mL, and ART-treated HIV patients with a viral load 20–1000 copies/mL. Table S2: Receiver operating characteristic analysis of the EV miRNA content as discriminant between HIV-negative and viremic patients. Table S3: Receiver operating characteristic analysis of the EV miRNA content as discriminant between nonviremic ART-naïve patients and viremic patients. Table S4: Receiver operating characteristic analysis of the EV miRNA content as discriminant between reference† patients and viremic patients. Table S5: Receiver operating characteristic analysis of the EV miRNA content as discriminant between HIV-negative and subgroups of viremic patients. Table S6: Receiver operating characteristic analysis of the EV miRNA content as discriminant between nonviremic ART-naïve patients and subgroups of viremic patients. Table S7: Receiver operating characteristic analysis of the EV miRNA content as discriminant between reference† patients and subgroups of viremic patients.
