# Supplementary material for: Vesicular MicroRNA as Potential Biomarkers of Viral Rebound
Source: Cells. 2022 Mar 2;11(5):859. doi: 10.3390/cells11050859 (PMC8909274; doi:10.3390/cells11050859)
Supplement: Supplementary file 1 [file cells-11-00859-s001.zip › cells-1573344-supplementary.pdf]

Supplementary files

Table S1: Demographic and clinical characteristics of the study participants

	Undetectable viral load						Viral load detectable ≥ 1000 copies/mL			Viral load detectable on ART		
	ART-naive with undetectable VL (n=12)	On ART more than 6 months with undetectable VL (n=128)	P-value	On ART less than 6 months with undetectable VL (n=10)	On ART more than 6 months with undetectable VL (n=128)	P-value	ART-naive (n = 14)	ART-treated (n = 36)	P-value	ART-treated VL ≥ 1000 copies/mL (n = 36)	ART-treated 20 ≤ VL < 1000 copies/mL (n = 32)	P-value
**Male: n (%)**	5 (41.7)	31 (24.2)	0.1861	4 (40.0)	31 (24.2)	0.2693	6 (42.9)	10 (27.3)	0.3047	10 (27.3)	12 (37.5)	0.3923
**Age (y)**	43 (28 – 52)	38 (32 – 45)	0.2300	34 (30 – 41)	38 (32 – 45)	0.3761	34 (26 – 40)	35 (30 – 38)	0.7007	35 (30 – 38)	35 (29 – 45)	0.2798
**< 20**	1 (8.3)	2 (1.6)		..	2 (1.6)		1 (7.1)	..		..	1 (3.1)	
**20 -29**	2 (16.7)	16 (12.5)		2 (20.00)	16 (12.5)		4 (28.6)	7 (19.4)		7 (19.4)	6 (18.8)	
**30 -39**	3 (25.0)	49 (38.3)	**0.0086**	5 (50.00)	49 (38.3)	0.6708	5 (35.7)	20 (55.6)	0.3268	20 (55.6)	12 (37.5)	0.1529
**40 - 49**	1 (8.3)	47 (36.7)		2 (20.00)	47 (36.7)		3 (21.4)	8 (22.2)		8 (22.2)	7 (21.9)	
**≥ 50**	5 (41.7)	14 (10.9)		1 (10.00)	14 (10.9)		1 (7.1)	1 (2.8)		1 (2.8)	6 (18.7)	
**HIV duration (month)**	24 (12 – 72)	55 (24 – 120)	0.1066	7 (3 – 16)	55 (2.00 – 10.00)	**0.0006**	12 (1 – 72)	36 (12 – 45)	0.8192	36 (12 – 45)	30 (9 – 96)	0.4987
**CD4 T cells/µL**	717 (466 – 875)	513 (386 – 755)	0.2040	415 (388 – 580)	513 (386 – 755)	0.1528	410 (304 – 657)	236 (139 – 372)	**0.0024**	236 (139 – 372)	484 (317 – 693)	**< 0.0001**
**CD8 T cells/µL**	650 (398 – 759)	736 (536 – 1093)	0.6081	1129 (953 – 1611)	736 (536 – 1093)	**0.0265**	920 (607 – 1063)	727 (526 – 941)	0.5158	727 (526 – 941)	898 (578 – 1176)	0.1836
**CD4/CD8**	1.2 (0.8 – 1.5)	0.7 (0.5 – 1.0)	**0.0038**	0.6 (0.4 – 0.9)	0.7 (0.5 – 1.0)	**0.0185**	0.5 (0.4 – 0.7)	0.3 (0.2 – 0.5)	**0.0284**	0.3 (0.2 – 0.5)	0.6 (0.4 – 0.9)	**0.0007**
**ART: n (%)**	68 (80.00%)	NA	..	NA	NA	..	NA	NA	..	NA	NA	..
**ART duration (month)**	NA NA	38 (21 – 96)	..	3 (2 – 4)	38 (21 – 96)	**0.0004**	NA NA	24 (9 – 42)	..	24 (9 – 42)	26 (7 – 70)	0.5312
**HIV-1 VL (copies/mL)**	NA NA	NA NA	.. ..	NA NA	NA NA	.. ..	25,258 (9,494 – 44,820)	23,544 (4,138 – 58,361)	0.7670	23,544 (4,138 – 58,361)	81 (54 – 247)	**0.0017**

Ranges indicated are interquartile (IQR); NA = not applicable; CD4 and CD8 counts are in cells per µL

HIV, ART, VL refer to human immunodeficiency virus or antiretroviral therapy status or viral load

Supplementary files

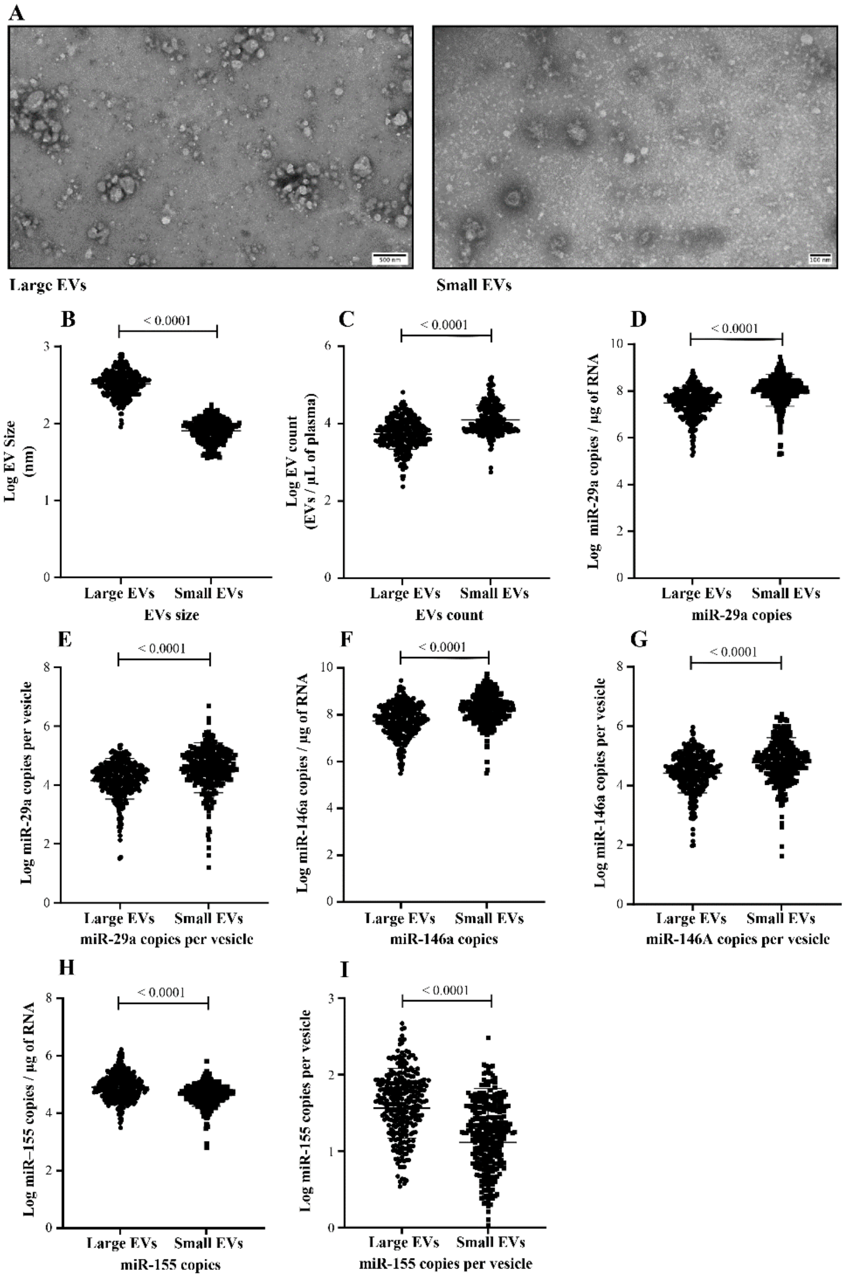

Figure S1: Extracellular vesicles characterization and miRNA content. Large and small EVs purified from study participants plasma were observed in transmission electronic microscopy (**A**), sized with dynamic light scaterring (**B**), counted in cytometry (**C**) and then their mature miR-29a (**D and E**), miR-146a (**F and G**), and miR-155 (**H and I**) content were analyzed in qPCR. Values were transformed to their logarithm. A paired t test was used for comparison.

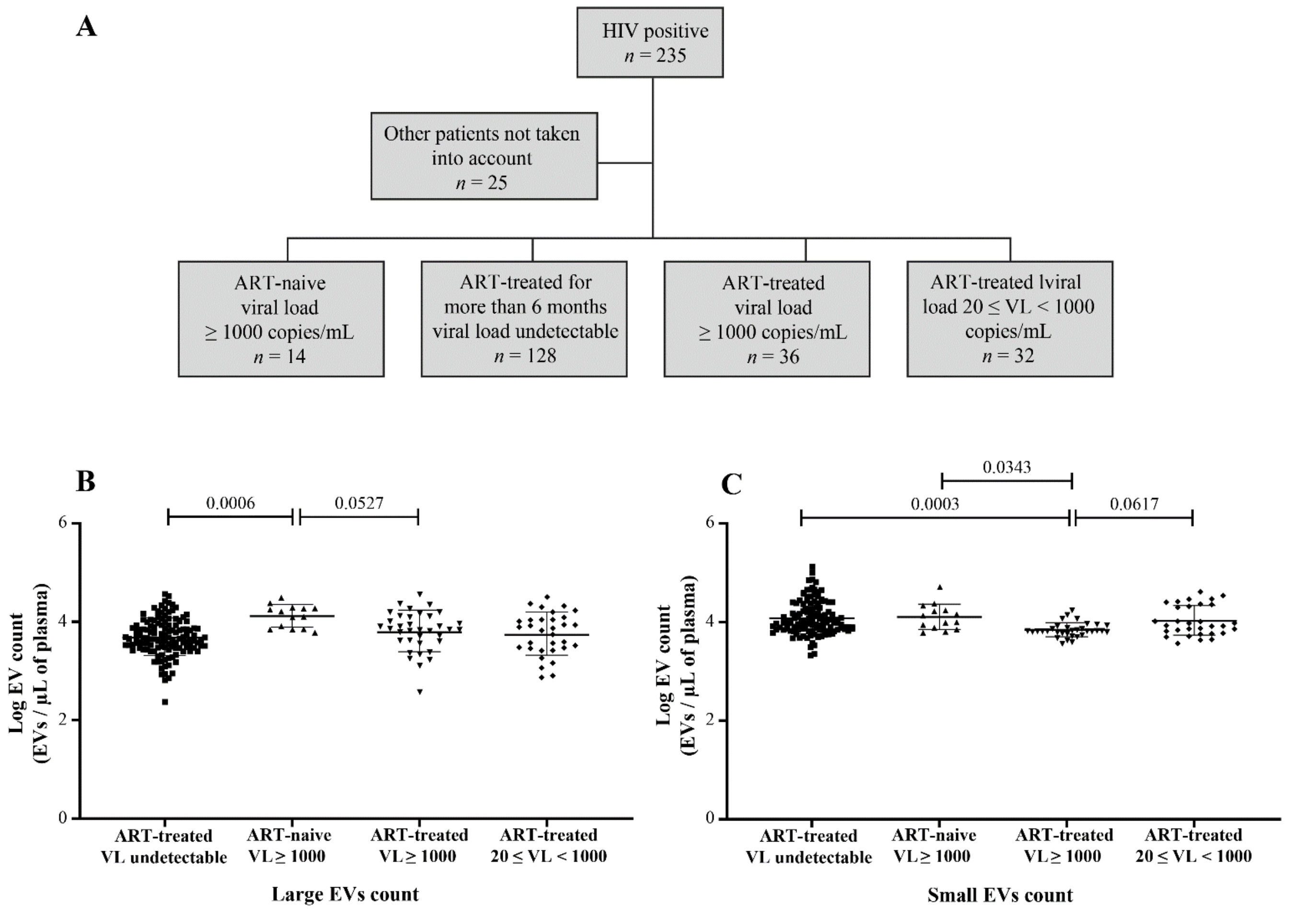

Figure S2: Extracellular vesicle analysis in cytometry. Large and small EVs purified from study participants plasma were staining with DID and CFSE and the particle number was counted in each fraction. Study participants were divided according to viral load level into (A): HIV positive antiretroviral (ART) treated for more than six months with undetectable viral load (VL) (ART-treated VL undetectable), ART-naive HIV positive with VL ≥ 1000 copies /mL (ART-naive VL ≥ 1000), ART-treated HIV positive with VL ≥ 1000 copies /mL (ART-treated VL≥ 1000), and HIV positive ART-treated with VL between 20 and 1000 copies/mL (ART-treated 20 ≤ VL < 1000). **(B)** Large EVs count and **(C)** small EVs count. An ordinary one-way ANOVA corrected for multiple comparisons using Tukey test was used for comparison between groups.

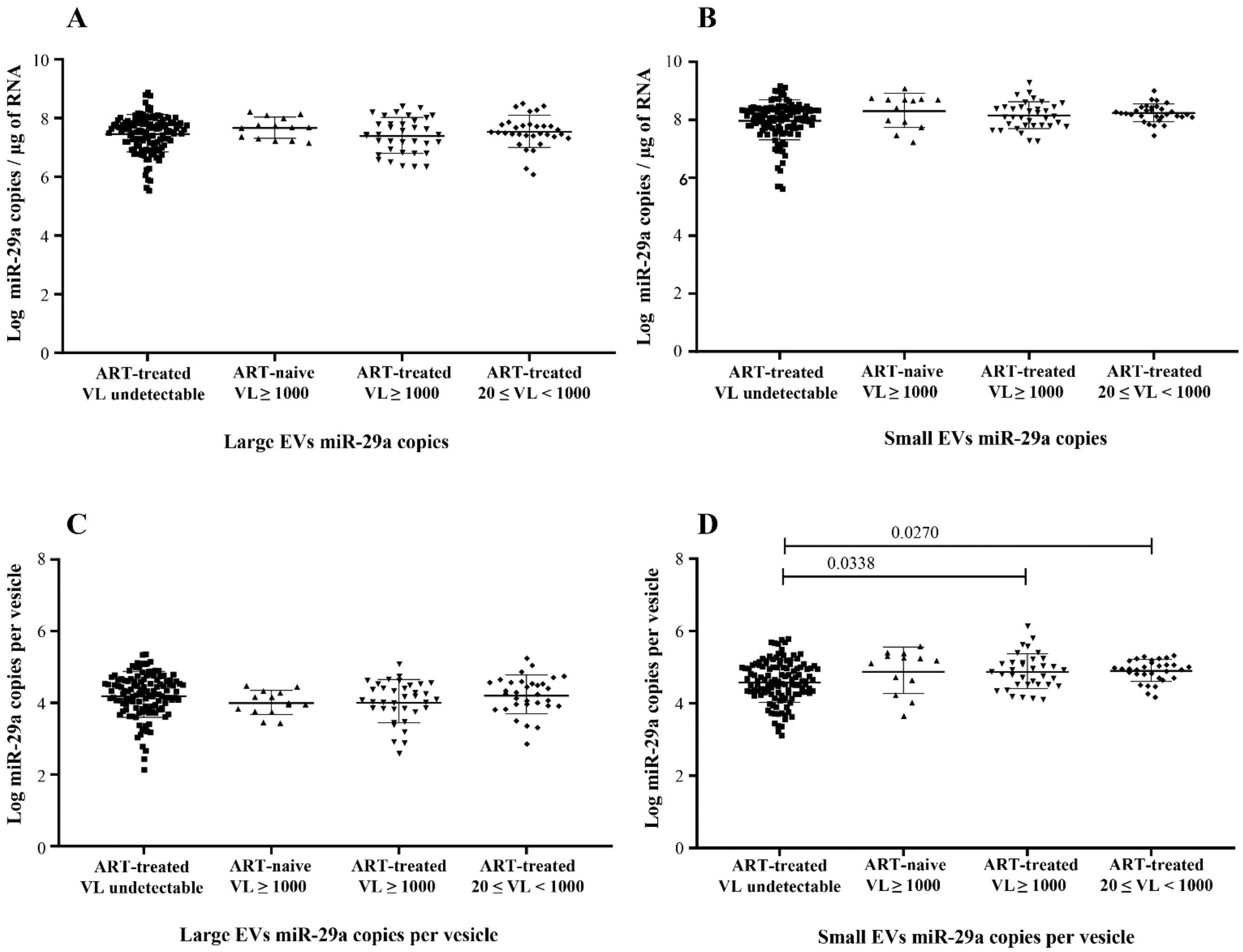

Figure S3: MicroRNA miR-29a content in large and small EVs in link with viral load status. Large **(A, C)** and small EVs **(B, D)** were purified from plasma of study participants divided into four groups as described in table S1. The mature miR-29a content expressed as copies per µg of total RNA **(A, B)** and copies per vesicle **(C, D)**. Values were transformed to their logarithm. An ordinary one-way ANOVA corrected for multiple comparisons using the Tukey test was used for comparison between group.

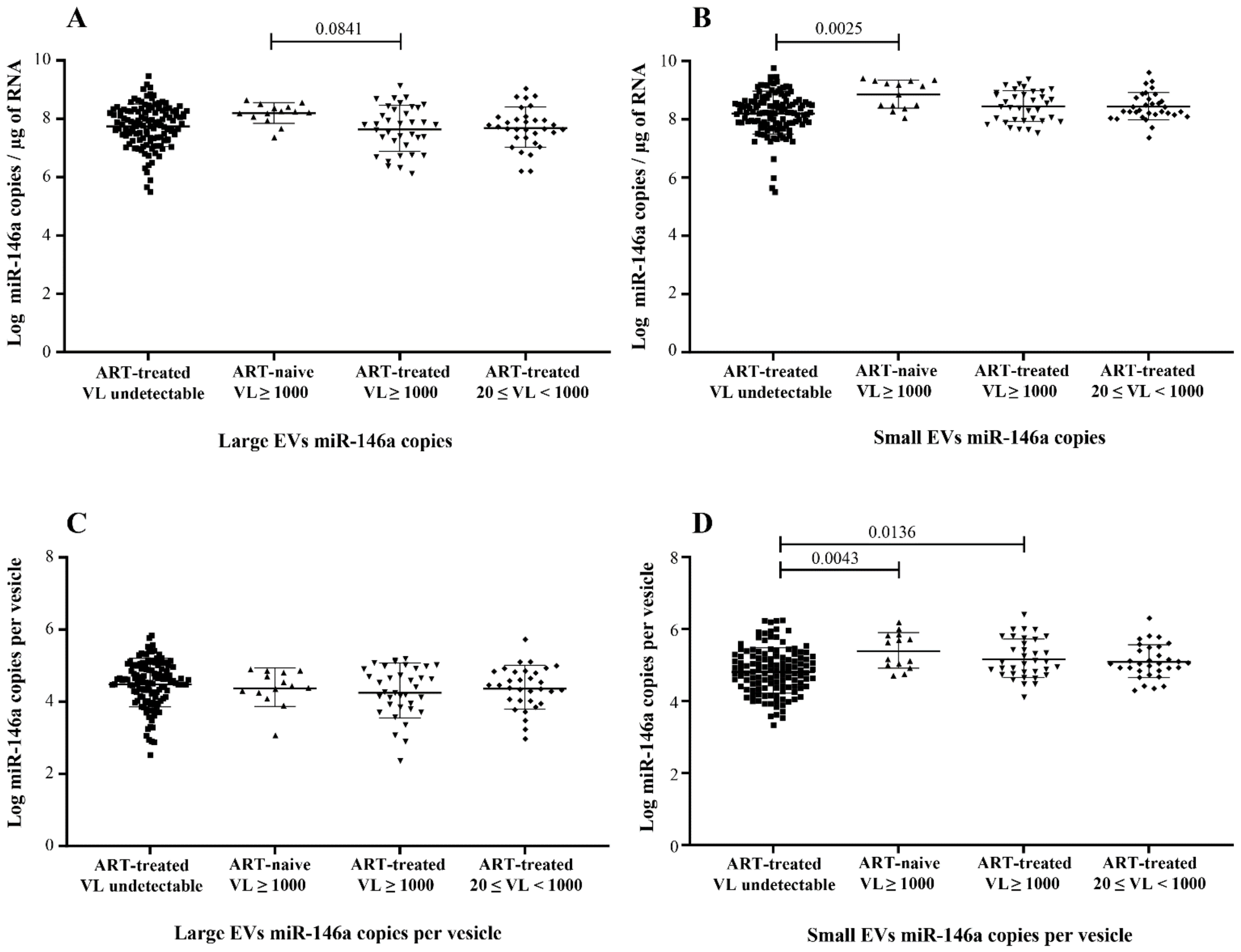

Figure S4: MicroRNA miR-146 content in large and small EVs in link with viral load status. Large **(A, C)** and small EVs **(B, D)** were purified from plasma of study participants divided into four groups as described in table S1. The mature miR-146 content expressed as copies per µg of total RNA **(A, B)** and copies per vesicle **(C, D)**. Values were transformed to their logarithm. An ordinary one-way ANOVA corrected for multiple comparisons using the Tukey test was used for comparison between group.

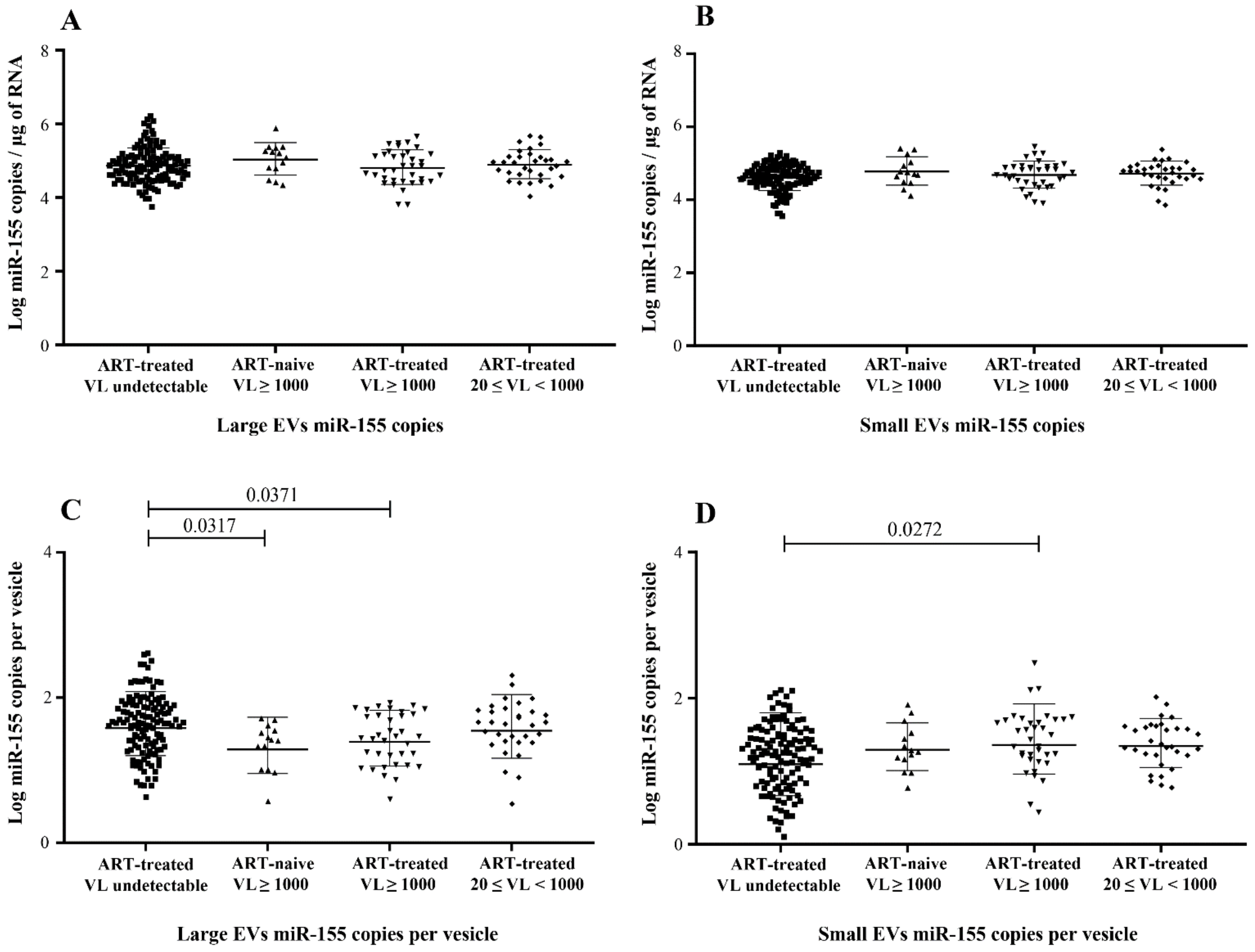

Figure S5: MicroRNA miR-155 content in large and small EVs in link with viral load status. Large **(A, C)** and small EVs **(B, D)** were purified from plasma of study participants divided into four groups as described in table S1. The mature miR-155 content expressed as copies per µg of total RNA **(A, B)** and copies per vesicle **(C, D)**. Values were transformed to their logarithm. An ordinary one-way ANOVA corrected for multiple comparisons using the Tukey test was used for comparison between group.

Supplementary files

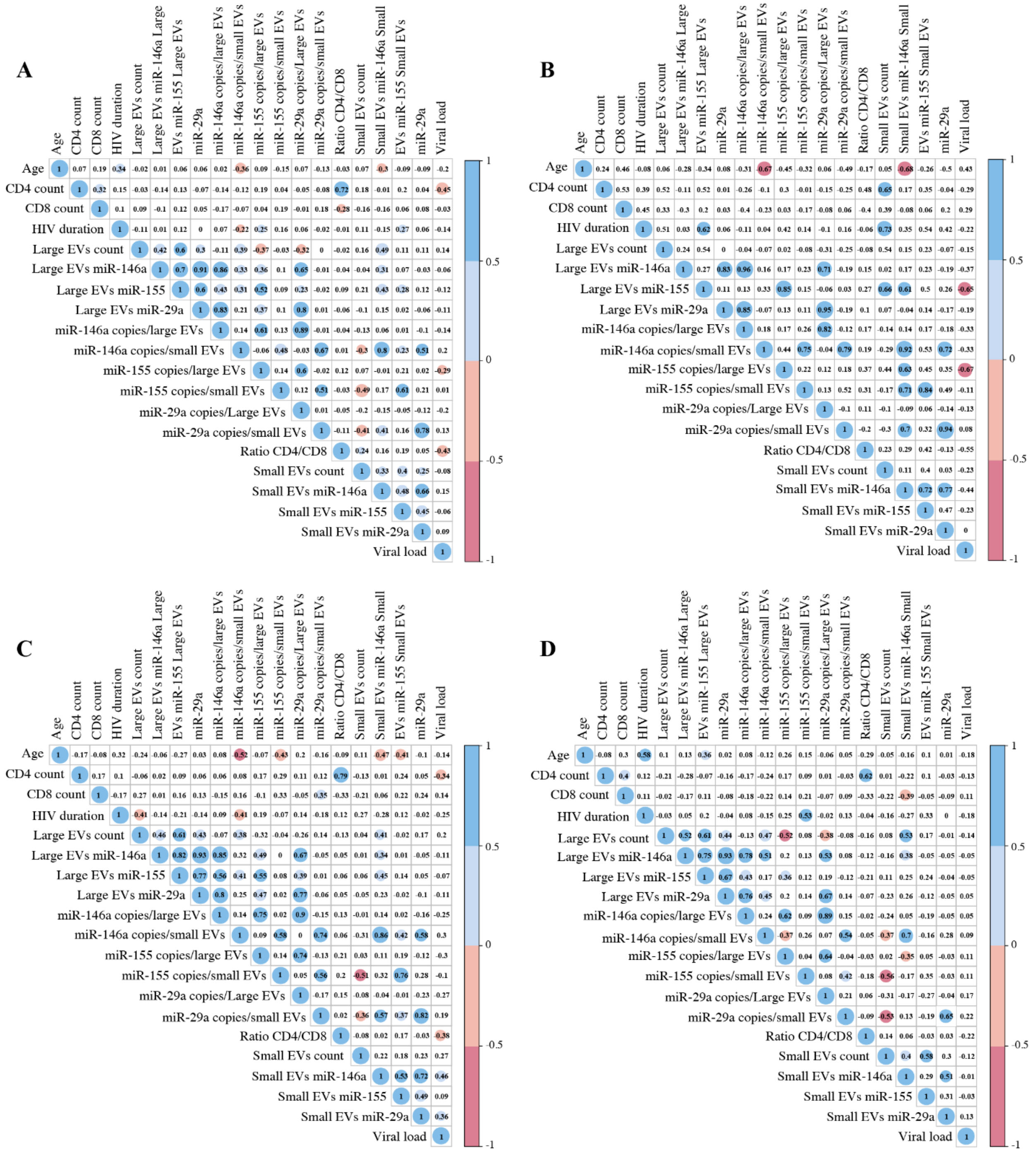

Figure S6: Correlation matrix between measurements on **A) viremic** HIV+ patients, **B) ART-naïve** HIV patients with viral load ≥ 1000 copies/mL, **C)** on **ART-treated** HIV patients with viral load ≥ 1000 copies/mL, **D)** on **ART-treated** HIV patients with viral load **20–1000** copies/mL. Circle size and color provide visualization of the relative strength of the correlation. Values are based on two-tailed Pearson correlational tests.

Table S2: Receiver operating characteristic analysis of EV miRNA content as discriminant between HIV-negative and viremic patients

	**HIV-negative as control**					
	**ART-naïve (≥ 1000 viral copies/mL)**		**ART-treated (≥ 1000 viral copies/mL)**		**ART-treated (20–1000 viral copies/mL)**	
	**AUC (95% CI)**	**P-value**	**AUC (95% CI)**	**P-value**	**AUC (95% CI)**	**P-value**
**miR-29a copies, large EV**	0.55 (0.39 – 0.71)	0.5437	0.57 (0.44 – 0.70)	0.2408	0.53 (0.41 – 0.66)	0.5828
**miR-29a copies, small EV**	**0.73 (0.54 – 0.92)**	**0.0092**	**0.66 (0.54 – 0.78)**	**0.0104**	**0.78 (0.69 – 0.88)**	**< 0.0001**
**miR-29a copies per large EV**	**0.78* (0.66 – 0.90)**	**0.0011**	**0.69* (0.58 – 0.80)**	**0.0021**	0.59 (0.46 – 0.71)	0.1710
**miR-29a copies per small EV**	**0.78 (0.62 – 0.94)**	**0.0017**	**0.79 (0.70 – 0.88)**	**< 0.0001**	**0.85 (0.76 – 0.93)**	**< 0.0001**
**miR-146a copies, large EV**	**0.76 (0.64 – 0.88)**	**0.0026**	0.51 (0.38 – 0.63)	0.8916	0.51 (0.38 – 0.64)	0.8633
**miR-146a copies, small EV**	**0.94 (0.88 – 1.00)**	**< 0.0001**	**0.77 (0.67 – 0.87)**	**< 0.0001**	**0.79 (0.70 – 0.89)**	**< 0.0001**
**miR-146a copies per large EV**	0.55 (0.40 – 0.70)	0.5437	0.56 (0.44 – 0.69)	0.2825	0.55 (0.43 – 0.68)	0.4170
**miR-146a copies per small EV**	**0.95 (0.90 – 0.99)**	**< 0.0001**	**0.88 (0.81 – 0.95)**	**< 0.0001**	**0.87 (0.80 – 0.95)**	**< 0.0001**
**miR-155 copies, large EV**	0.58 (0.40 – 0.76)	0.3407	0.57 (0.44 – 0.69)	0.2627	0.50 (0.38– 0.63)	0.9425
**miR-155 copies, small EV**	0.64 (0.42 – 0.82)	0.1087	0.58 (0.45 – 0.71)	0.1699	**0.69 (0.57 – 0.81)**	**0.0036**
**miR-155 copies per large EV**	**0.84* (0.75 – 0.93)**	**< 0.0001**	**0.73* (0.63 – 0.83)**	**0.0001**	**0.63* (0.51 – 0.74)**	**0.0455**
**miR-155 copies per small EV**	**0.72 (0.59 – 0.86)**	**0.0094**	**0.76 (0.66 – 0.86)**	**< 0.0001**	**0.75 (0.65 – 0.85)**	**< 0.0001**

ART: antiretroviral therapy status, AUC: area under the curve, CI: confidence interval

* Values are higher in the control group

Table S3: Receiver operating characteristic analysis of EV miRNA content as discriminant between non-viremic ART-naïve patients and viremic patients

	**ART-naive non-viremic patients as control**					
	**ART-naive (≥ 1000 viral copies/mL)**		**ART-treated (≥ 1000 viral copies/mL)**		**ART-treated (20–1000 viral copies/mL)**	
	**AUC (95% CI)**	**P-value**	**AUC (95% CI)**	**P-value**	**AUC (95% CI)**	**P-value**
**miR-29a copies, large EV**	0.58 (0.35 – 0.80)	0.5037	**0.71* (0.55 – 0.86)**	**0.0394**	0.63 (0.44 – 0.83)	0.17066
**miR-29a copies, small EV**	0.66 (0.43 – 0.90)	0.1734	0.54 (0.35 – 0.72)	0.6898	0.64 (0.46 – 0.83)	0.1568
**miR-29a copies per large EV**	0.65 (0.42 – 0.89)	0.1889	0.53 (0.33 – 0.74)	0.7210	0.56 (0.36 – 0.76)	0.5444
**miR-29a copies per small EV**	**0.96 (0.89 – 1.00)**	**0.0003**	**0.86 (0.73 – 0.98)**	**0.0004**	**0.92 (0.83 – 1.00)**	**< 0.0001**
**miR-146a copies, large EV**	0.55 (0.32 – 0.78)	0.6807	**0.76* (0.62 – 0.89)**	**0.0084**	**0.79* (0.66 – 0.92)**	**0.0034**
**miR-146a copies, small EV**	0.58 (0.34 – 0.81)	0.5037	0.64 (0.49 – 0.80)	0.1432	**0.77* (0.63 – 0.91)**	**0.0068**
**miR-146a copies per large EV**	061 (0.38 – 0.83)	0.3545	0.63 (0.44 – 0.81)	0.1904	0.63 (0.43 – 0.83)	0.1937
**miR-146a copies per small EV**	**0.80 (0.62 – 0.97)**	**0.0118**	0.67 (0.51 – 0.83)	0.0910	0.64 (0.47 – 0.81)	0.1937
**miR-155 copies, large EV**	**0.78* (0.60– 0.96)**	**0.0167**	**0.82* (0.70 – 0.94)**	**0.0010**	**0.83* (0.71 – 0.95)**	**0.0007**
**miR-155 copies, small EV**	0.52 (0.29 – 0.75)	0.8370	0.51 (0.32 – 0.69)	0.9431	0.53 (0.33 – 0.73)	0.7518
**miR-155 copies per large EV**	**0.75* (0.56 – 0.94)**	**0.0281**	0.68 (0.51– 0.84)	0.0668	0.54 (0.35 – 0.73)	0.6542
**miR-155 copies per small EV**	**0.90 (0.76 – 1.00)**	**0.0010**	**0.94 (0.86 – 1.00)**	**< 0.0001**	**0.92 (0.82 – 1.00)**	**< 0.0001**

ART: antiretroviral therapy status, AUC: area under the curve, CI: confidence interval

Table S4: Receiver operating characteristic analysis of EV miRNA content as discriminant between reference† patients and viremic patients

	Reference patients as control					
	ART-naive (VL ≥ 1000 copies/mL)		ART-treated (VL ≥ 1000 copies/mL)		ART-treated (20 ≤ VL < 1000 copies/mL)	
	AUC (95% CI)	P value	AUC (95% CI)	P value	AUC (95% CI)	P value
**miR-29a copies, large EV**	0.63 (0.40 – 0.87)	0.2659	0.55 (0.35 – 0.76)	0.5943	0.57 (0.34 – 0.80)	0.4920
**miR-29a copies, small EV**	0.59 (0.34 – 0.85)	0.4568	0.57 (0.40 – 0.74)	0.4949	0.53 (0.31 – 0.74)	0.7847
**miR-29a copies per large EV**	0.51 (0.24 – 0.77)	0.9533	0.50 (0.27 – 0.74)	0.9575	0.57 (0.34 – 0.80)	0.4970
**miR-29a copies per small EV**	**0.93 (0.82 – 1.00)**	**0.0015**	**0.71 (0.51 – 0.91)**	**0.0530**	**0.77 (0.58 – 0.98)**	**0.0141**
**miR-146a copies, large EV**	**0.81 (0.62 – 0.99)**	**0.0118**	0.57 (0.38 – 0.77)	0.4721	0.59 (0.38 – 0.81)	0.3818
**miR-146a copies, small EV**	**0.81 (0.63 – 0.98)**	**0.0118**	0.62 (0.43 – 0.81)	0.2630	0.65 (0.42 – 0.87)	0.1693
**miR-146a copies per large EV**	0.57 (0.32 – 0.82)	0.5582	0.53 (0.32 – 0.74)	0.7900	0.56 (0.34 – 0.77)	0.5950
**miR-146a copies per small EV**	**0.86 (0.70 – 1.00)**	**0.0034**	**0.78 (0.61 – 0.94)**	**0.0085**	**0.75 (0.56 – 0.93)**	**0.0196**
**miR-155 copies, large EV**	**0.82 (0.63– 0.99)**	**0.0117**	**0.72 (0.55– 0.90)**	**0.0394**	**0.82 (0.67 – 0.96)**	**0.0053**
**miR-155 copies, small EV**	**0.59 (0.35 – 0.82)**	**0.4884**	0.57 (0.40 – 0.75)	0.4959	0.67 (0.48 – 0.86)	0.1252
**miR-155 copies per large EV**	0.58 (0.31 – 0.86)	0.4823	0.50 (0.25 – 0.75)	0.9788	0.58 (0.33 – 0.82)	0.4479
**miR-155 copies per small EV**	**0.84 (0.67 – 1.00)**	**0.0095**	**0.90 (0.80 – 1.00)**	**0.0005**	**0.87 (0.79 – 0.99)**	**0.0019**

†non-viremic ART-treated for more than six months, CD4 cell count ≥ 500, CD8 cell count < 500

ART: antiretroviral therapy status, CI: confidence interval, VL: viral load

Table S5: Receiver operating characteristic analysis of EV miRNA content as discriminant between HIV-negative and sub groups of viremic patients

	**HIV negative as control**							
	**Sex workers**		**Female from general population**		**Men who have sex with men**		**Men from general population**	
	**AUC (95% CI)**	**P value**	**AUC (95% CI)**	**P value**	**AUC (95% CI)**	**P value**	**AUC (95% CI)**	**P value**
**miR-29a copies, large EV**	**0.68* (0.55 – 0.80)**	**0.0062**	0.56 (0.42 – 0.70)	0.3793	0.50 (0.35 – 0.65)	0.9908	0.63 (0.43 – 0.83)	0.2123
**miR-29a copies, small EV**	**0.69 (0.57 – 0.81)**	**0.0042**	**0.64 (0.51 – 0.78)**	**0.0331**	**0.83 (0.74 – 0.93)**	**< 0.0001**	**0.78 (0.60 – 0.96)**	**0.0119**
**miR-29a copies per large EV**	**0.71* (0.60 – 0.83)**	**0.0011**	0.57 (0.43 – 0.71)	0.3085	**0.70* (0.57 – 0.83)**	**0.0095**	**0.71* (0.52 – 0.90)**	**0.0441**
**miR-29a copies per small EV**	**0.79 (0.69 – 0.88)**	**< 0.0001**	**0.74 (0.62 – 0.87)**	**0.0004**	**0.89 (0.82 – 0.97)**	**< 0.0001**	**0.91 (0.84 – 0.98)**	**0.0004**
**miR-146a copies, large EV**	0.62 (0.48 – 0.75)	0.0743	0.61 (0.48 – 0.74)	0.1094	0.58 (0.42 – 0.73)	0.3211	**0.76 (0.60 – 0.91)**	**0.0139**
**miR-146a copies, small EV**	**0.68 (0.56 – 0.81)**	**0.0053**	**0.79 (0.70 – 0.89)**	**< 0.0001**	**0.95 (0.90 – 1.0)**	**< 0.0001**	**0.94 (0.86 – 1.0)**	**< 0.0001**
**miR-146a copies per large EV**	**0.64* (0.52 – 0.77)**	**0.0297**	0.52 (0.39 – 0.66)	0.7481	0.57 (0.42 – 0.73)	0.3296	0.51 (0.29 – 0.72)	0.9432
**miR-146a copies per small EV**	**0.83 (0.74 – 0.91)**	**< 0.0001**	**0.89 (0.82 – 0.96)**	**< 0.0001**	**0.95 (0.90 – 1.0)**	**< 0.0001**	**0.96 (0.92 – 1.0)**	**< 0.0001**
**miR-155 copies, large EV**	**0.67* (0.55 – 0.80)**	**0.0072**	0.55 (0.42 – 0.69)	0.4372	0.53 (0.37– 0.68)	0.7498	**0.79 (0.65 – 0.93)**	**0.0055**
**miR-155 copies, small EV**	0.60 (0.46 – 0.74)	0.1266	0.60 (0.46 – 0.73)	0.1545	**0.70 (0.56 – 0.84)**	**0.0092**	**0.76 (0.56 – 0.97)**	**0.0113**
**miR-155 copies per large EV**	**0.72* (0.61 – 0.82)**	**0.0008**	0.62 (0.48 – 0.75)	0.0846	**0.74* (0.63 – 0.85)**	**0.0016**	0.66 (0.47 – 0.84)	0.1345
**miR-155 copies per small EV**	**0.72 (0.61 – 0.83)**	**0.0008**	**0.77 (0.67 – 0.87)**	**< 0.0001**	**0.79 (0.68 – 0.91)**	**0.0002**	**0.79 (0.77 – 0.94)**	**0.0062**

HIV: human immunodeficiency virus, AUC: area under the curve, CI: confidence interval

* Values are higher in the control

Table S6: Receiver operating characteristic analysis of EV miRNA content as discriminant between non-viremic ART-naïve patients and sub groups of viremic patients

	ART-naive, undetectable viral load							
	Sex workers		Female from general population		Men who have sex with men		Men from general population	
	AUC (95% CI)	P value	AUC (95% CI)	P value	AUC (95% CI)	P value	AUC (95% CI)	P value
**miR-29a copies, large EV**	**0.75* (0.60 – 0.91)**	**0.0113**	0.58 (0.39 – 0.77)	0.4289	0.61 (0.40 – 0.82)	0.3304	0.52 (0.26 – 0.78)	0.8870
**miR-29a copies, small EV**	0.55 (0.38 – 0.73)	0.6447	0.53 (0.34 – 0.71)	0.8289	0.66 (0.46 – 0.86)	0.1494	0.65 (0.36 – 0.93)	0.2831
**miR-29a copies per large EV**	0.57 (0.36 – 0.77)	0.5040	0.56 (0.37 – 0.76)	0.5228	0.52 (0.30 – 0.76)	0.7765	0.53 (0.27 – 0.78)	0.8312
**miR-29a copies per small EV**	**0.86 (0.72 – 0.99)**	**0.0006**	**0.81 (0.67 – 0.95)**	**0.0032**	**0.96 (0.91 – 1.0)**	**< 0.0001**	**0.95 (0.85 – 1.0)**	**0.0018**
**miR-146a copies, large EV**	**0.83* (0.71 – 0.95)**	**0.0009**	0.67 (0.50 – 0.84)	0.0884	0.70 (0.51 – 0.88)	0.0680	0.52 (0.23 – 0.80)	0.9093
**miR-146a copies, small EV**	**0.77* (0.72 – 0.91)**	**0.0080**	**0.76* (0.61 – 0.91)**	**0.0097**	0.61 (0.42 – 0.81)	0.2917	0.65 (0.38 – 0.91)	0.2555
**miR-146a copies per large EV**	0.69 (0.52 – 0.87)	0.0513	0.54 (0.34 – 0.74)	0.6924	0.62 (0.40 – 0.83)	0.2735	0.57 (0.32 – 0.82)	0.5697
**miR-146a copies per small EV**	0.51 (0.32 – 0.70)	0.8916	0.67 (0.49 – 0.85)	0.1111	**0.87 (0.74 – 1.0)**	**0.0009**	**0.89 (0.75 – 1.00)**	**0.0034**
**miR-155 copies, large EV**	**0.90* (0.81 – 0.99)**	**< 0.0001**	**0.75* (0.60 – 0.90)**	**0.0137**	**0.84* (0.70 – 0.98)**	**0.0017**	0.51 (0.22 – 0.79)	0.9697
**miR-155 copies, small EV**	0.51 (0.32 – 0.70)	0.9113	0.52 (0.32 – 0.72)	0.8195	0.56 (0.35 – 0.78)	0.5565	0.69 (0.45 – 0.92)	0.1552
**miR-155 copies per large EV**	0.65 (0.48 – 0.83)	0.1190	0.56 (0.37 – 0.74)	0.5632	0.67 (0.47 – 0.87)	0.1137	0.61 (0.36 – 0.86)	0.3938
**miR-155 copies per small EV**	**0.91 (0.80 – 1.0)**	**0.0001**	**0.91 (0.79 – 1.00)**	**0.0001**	**0.95 (0.87 – 1.00)**	**< 0.0001**	**0.92 (0.80 – 1.00)**	**0.0019**

ART: antiretroviral therapy, AUC: area under the curve, CI: confidence interval

* Values are higher in the control

Table S7: Receiver operating characteristic analysis of EV miRNA content as discriminant between reference† patients and sub groups of viremic patients

	Reference patients as control							
	Sex workers		Female from general population		Men who have sex with men		Men from general population	
	**AUC (95% CI)**	**P value**	**AUC (95% CI)**	**P value**	**AUC (95% CI)**	**P value**	**AUC (95% CI)**	**P value**
**miR-29a copies, large EV**	0.55 (0.33 – 0.78)	0.6172	0.63 (0.43 – 0.84)	0.2247	0.58 (0.34 – 0.81)	0.4913	0.72 (0.49 – 0.95)	0.1025
**miR-29a copies, small EV**	0.50 (0.30 – 0.69)	0.9999	0.54 (0.34 – 0.72)	0.7195	0.60 (0.38 – 0.82)	0.3833	0.56 (0.26 – 0.86)	0.6569
**miR-29a copies per large EV**	0.53 (0.30 – 0.76)	0.7786	0.59 (0.36 – 0.81)	0.4315	0.52 (0.26 – 0.77)	0.8905	0.53 (0.26 – 0.81)	0.8065
**miR-29a copies per small EV**	0.62 (0.39 – 0.84)	0.2743	0.65 (0.46 – 0.83)	0.1713	**0.77 (0.57 – 0.96)**	**0.0214**	**0.81 (0.61 – 1.00)**	**0.0318**
**miR-146a copies, large EV**	0.53 (0.31 – 0.74)	0.7786	0.66 (0.47 – 0.85)	0.1414	0.64 (0.43 – 0.85)	0.2329	**0.80 (0.60 – 1.00)**	**0.0275**
**miR-146a copies, small EV**	0.51 (0.30 – 0.72)	0.8976	0.61 (0.38 – 0.84)	0.3049	**0.86 (0.72 – 0.99)**	**0.0018**	**0.82 (0.63 – 1.00)**	**0.0179**
**miR-146a copies per large EV**	0.55 (0.32 – 0.77)	0.5582	0.62 (0.41 – 0.83)	0.2738	0.54 (0.31 – 0.77)	0.7136	0.59 (0.32 – 0.85)	0.5136
**miR-146a copies per small EV**	0.66 (0.44 – 0.88)	0.1306	**0.74 (0.55 – 0.95)**	**0.0219**	**0.91 (0.80 – 1.00)**	**0.0004**	**0.90 (0.76 – 1.00)**	**0.0033**
**miR-155 copies, large EV**	0.51 (0.30 – 0.74)	0.8636	**0.80 (0.64 – 0.96)**	**0.0085**	**0.77 (0.59 – 0.96)**	**0.0221**	**0.89 (0.74 – 1.00)**	**0.0043**
**miR-155 copies, small EV**	0.53 (0.34 – 0.71)	0.8027	0.52 (0.32 – 0.72)	0.8374	0.59 (0.37 – 0.81)	0.4089	0.72 (0.47 – 0.97)	0.1025
**miR-155 copies per large EV**	0.51 (0.27 – 0.75)	0.9006	0.57 (0.34 – 0.80)	0.5158	0.53 (0.27 – 0.80)	0.7831	0.58 (0.31 – 0.86)	0.5136
**miR-155 copies per small EV**	**0.84 (0.70 – 0.98)**	**0.0036**	**0.88 (0.76 – 1.00)**	**0.0012**	**0.89 (0.78 – 1.00)**	**0.0014**	**0.89 (0.73 – 1.00)**	**0.0071**

†non-viremic ART-treated for more than six months, CD4 cell count ≥ 500, CD8 cell count < 500

AUC: area under the curve, CI: confidence interval, VL: viral load
